# Insights Into Pneumococcal Pneumonia Using Lung Aspirates and Nasopharyngeal Swabs Collected From Pneumonia Patients in The Gambia

**DOI:** 10.1093/infdis/jiaa186

**Published:** 2020-04-22

**Authors:** Eileen M Dunne, Yinglei Hua, Rasheed Salaudeen, Ilias Hossain, Malick Ndiaye, Belinda D Ortika, E Kim Mulholland, Jason Hinds, Sam Manna, Grant A Mackenzie, Catherine Satzke

**Affiliations:** 1 Infection and Immunity, Murdoch Children’s Research Institute, Parkville, Australia; 2 Department of Paediatrics, The University of Melbourne, Parkville, Australia; 3 Department of Microbiology and Immunology, The University of Melbourne at the Peter Doherty Institute for Infection and Immunity, Parkville, Australia; 4 Medical Research Council Unit The Gambia at the London School of Hygiene and Tropical Medicine, Fajara, The Gambia; 5 Department of Infectious Disease Epidemiology, London School of Hygiene & Tropical Medicine, London, United Kingdom; 6 Institute for Infection and Immunity, St. George’s University of London, London, United Kingdom; 7 BUGS Bioscience, London Bioscience Innovation Centre, London, United Kingdom; 8 Department of Disease Control, London School of Hygiene & Tropical Medicine, London, United Kingdom

**Keywords:** lung, nasopharynx, pneumococcus, pneumonia, *Streptococcus pneumoniae*

## Abstract

**Background:**

We investigated the pathogenesis of pneumococcal pneumonia using clinical specimens collected for pneumonia surveillance in The Gambia.

**Methods:**

Lung aspirates and nasopharyngeal swabs from 31 patients were examined by culture, quantitative polymerase chain reaction (qPCR), whole genome sequencing, serotyping, and reverse-transcription qPCR.

**Results:**

Five lung aspirates cultured pneumococci, with a matching strain identified in the nasopharynx. Three virulence genes including *ply* (pneumolysin) were upregulated >20-fold in the lung compared with the nasopharynx. Nasopharyngeal pneumococcal density was higher in pediatric pneumonia patients compared with controls (*P* < .0001).

**Conclusions:**

Findings suggest that changes in pneumococcal gene expression occurring in the lung environment may be important in pathogenesis.

## Teaser

We investigated the pathogenesis of pneumococcal pneumonia using clinical samples. Results demonstrated that pneumococci in the lung originated from the nasopharynx and suggested that changes in pneumococcal gene expression in the lung are a feature of pneumococcal pneumonia.

*Streptococcus pneumoniae* (the pneumococcus) is a common cause of community-acquired pneumonia, a major public health problem that primarily affects young children and the elderly. Pneumococcal pneumonia is believed to occur when pneumococci colonizing the upper respiratory tract are aspirated into the lung, where a failure to clear the bacteria leads to replication and triggers a damaging immune response. Supporting clinical data are limited because lung aspirates are not routinely collected in most settings; however, a previous study from The Gambia found that 85% of pediatric pneumonia patients with pneumococci identified in lung aspirates had the same serotype detected in a nasopharyngeal swab [1]. Studies have found higher pneumococcal loads in the nasopharynx in children with pneumonia compared with healthy controls, suggesting that increased pneumococcal density in the nasopharynx may facilitate bacterial spread to the lung, or alternatively, may be due to pneumonia [2, 3]. The processes by which pneumococci transition from a colonizing to a pathogenic state are not well understood.

Pneumococci possess a variety of virulence factors, including polysaccharide capsule, the toxin pneumolysin, adhesins, and surface proteins that allow them to cause disease [4]. After examining pneumococcal gene expression in a range of in vitro conditions designed to mimic host microenvironments, Aprianto et al [5] identified 498 conditionally expressed genes, highlighting the dynamic transcriptional ability of the pneumococcus. Experiments using animal models demonstrated that the pneumococcus modifies its gene expression in response to different niches within the host [6, 7]. To date, only a single published study has examined pneumococcal gene expression in clinical samples. Using nasopharyngeal swabs collected from healthy children, Sakai et al [8] found different levels of expression for 11 pneumococcal genes, demonstrating the feasibility of evaluating pneumococcal gene expression directly from clinical samples.

Using a unique set of samples collected as part of pneumonia surveillance in The Gambia, we sought to investigate the pathogenesis of pneumococcal pneumonia by comparing pneumococcal loads in the nasopharynx of pneumonia patients and healthy carriers, conducting whole genome sequencing of paired pneumococcal isolates obtained from the nasopharynx and lung of pneumonia patients, and analyzing pneumococcal gene expression directly from nasopharyngeal swabs and lung aspirates.

## METHODS

Detailed methods are provided in the Supplementary Information. This study was nested within population-based surveillance for suspected pneumonia, septicemia, and meningitis among patients aged 2 months or greater in the Basse Health and Demographic surveillance system in The Gambia, as described previously [9]. All patients with suspected pneumonia who had a lung aspiration from April 2015 to May 2016 were included in this study. Nasopharyngeal swabs were collected from pneumonia patients and community controls. For all swabs and lung aspirates, an aliquot for ribonucleic acid (RNA) analysis was prepared by adding 2-fold volume of RNAprotect Bacteria Reagent (QIAGEN) to the clinical sample.

The surveillance project and this substudy were approved by The Gambia Government/Medical Research Council Joint Ethics Committee (approval numbers 1087 and L2016.09v2). Patients or their parent or guardian provided written informed consent for enrolment in the surveillance project and lung aspiration.

### Laboratory Procedures

In brief, nasopharyngeal swab samples underwent deoxyribonucleic acid (DNA) extraction and *lytA* real-time quantitative polymerase chain reaction (qPCR) to detect and quantify pneumococci. Pneumococcal density data are reported as genome equivalents/mL (GE/mL). Molecular serotyping by microarray was conducted on nasopharyngeal swabs. Pneumococci isolated from the lung and nasopharynx were serotyped by traditional antibody-based methods and whole genome sequencing conducted using the MiSeq platform. Expression of 9 pneumococcal genes selected from the literature was examined by reverse-transcription qPCR after RNA extraction from clinical specimens. Genes and primer sequences are provided in Supplementary Table 1.

Data analysis was performed using STATA version 15.1 and GraphPad Prism version 7.03. Categorical data were compared using the χ ^2^ test. Pneumococcal density data were log_10_ transformed before analysis and examined using non-parametric methods. Densities were compared using the Mann-Whitney test, and multivariable quantile regression models included age, sex, and Pneumococcal conjugate vaccine (PCV) status as variables selected a priori.

## RESULTS

**Figure 1.**
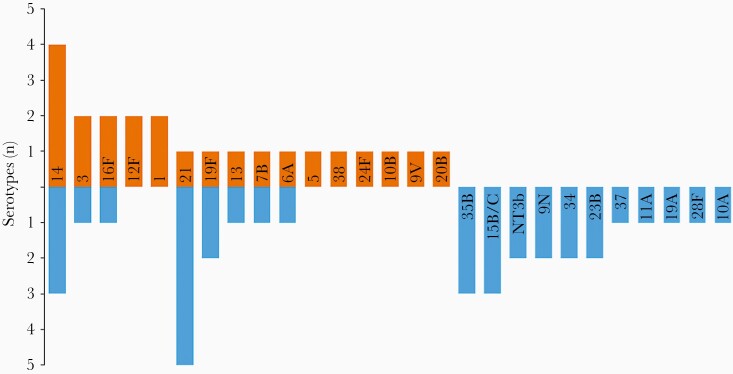
Mirror plot of pneumococcal serotypes identified in nasopharyngeal swabs of children <5 years of age hospitalized with pneumonia (orange bars) compared with pneumococcal serotypes found in nasopharyngeal swabs collected from community controls (blue bars). NT3b refers to a lineage of nonencapsulated *Streptococcus pneumoniae*.

Samples from 31 pneumonia patients enrolled in surveillance were included in this study. Patient ages ranged from 2 months to 73 years, with a median of 3.8 years (interquartile range [IQR], 1.7–7.0). The majority were children, with 20 (65%) aged <5 years, 6 (19%) from 5 to 18 years, and 5 (16%) >18 years, and 20 of 31 (65%) were male. Examination of nasopharyngeal swabs from patients of all ages found that 27 (87%) contained pneumococci. We compared the prevalence, density, and serotypes of pneumococci in the nasopharynx of patients <5 years of age with community controls. Participant characteristics are shown in Supplementary Table 2. Pneumococcal prevalence was similar in pneumonia patients (18 of 20; 90%) and community controls (21 of 22, 96%, *P* = .493, χ ^2^ test). Among children with pneumococci detected in the nasopharynx, median density was higher in pneumonia patients (7.77 log_10_ GE/mL; IQR, 7.33–7.98) compared with controls (6.05; IQR, 5.67–6.47; *P* < .0001). When adjusted for age, sex, and PCV status, the difference remained significant, with a coefficient (difference between medians) of 1.61 log_10_ GE/mL (95% confidence interval, 0.96–2.26; *P* < .001). Serotypes identified in nasopharyngeal swabs from pneumonia patients and controls are shown in Figure 1. Eight serotypes were found in both participant groups, with 8 additional serotypes found only in pneumonia patients and 11 only found in community controls.

Culture of lung aspirates from 31 pneumonia patients of all ages identified pneumococci as the causative organism in 5 patients (serotypes 3, 14, 1, 12F, and 32A; n = 1 each) and *Staphylococcus aureus* in 1. All 5 patients who had pneumococci cultured from the lung aspirate had the same serotype identified in their nasopharyngeal swab. Pneumococcal *lytA* qPCR was conducted for the lung aspirates with sufficient volume remaining for analysis (n = 25). Ten (40%) lung aspirates tested positive by qPCR, including the 5 samples that were also positive by culture. Median pneumococcal density was higher in the culture-positive lung aspirates (5.74 log_10_ GE/mL; IQR, 4.93–5.93) compared with the culture-negative lung aspirates (3.32 log_10_ GE/mL; IQR, 2.86–3.88; *P* = .008).

To identify within-host genomic changes, in particular DNA mutations that may contribute to pneumococcal pathogenesis, whole genome sequencing was conducted on the 5 paired pneumococcal isolates from the lung and nasopharynx of the same patient. For all 5 patients, the isolate from the nasopharynx was the same as that from the lung, with >99% sequence similarity and matching multilocus sequence types (Supplementary Table 3). Three mutations were identified in isolates from lung aspirates (Supplementary Table 4).

**Figure 2.**
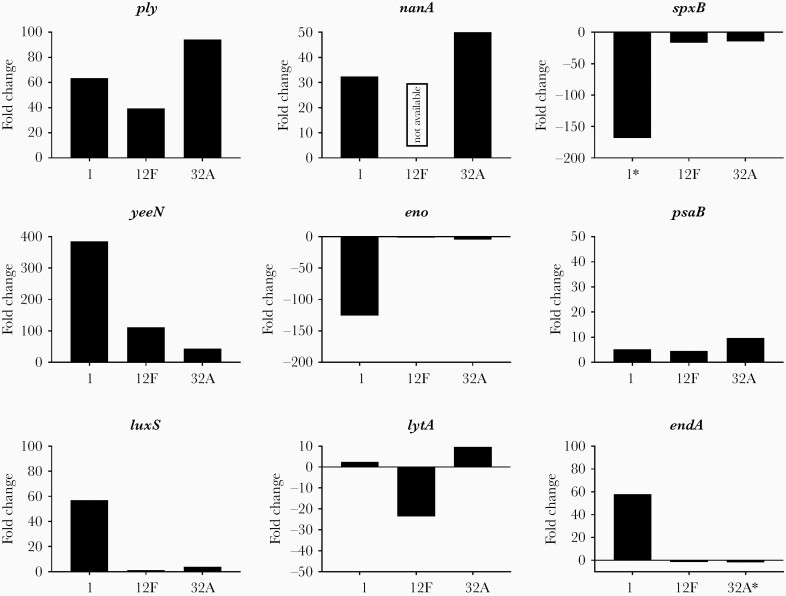
Differential expression of pneumococcal genes in lung aspirates compared with nasopharyngeal swabs collected from the same patient by reverse-transcription quantitative polymerase chain reaction. Fold change in gene expression was derived using the 2^−ΔΔCt^ method with *gyrA* as the reference gene. Each bar represents data from one gene in 1 set of patient samples. * indicates an imputed Cycle threshold (Ct) value of 40 used for the lung aspirate sample. For serotype 12F, data for *nanA* are not available because no transcripts were detected in either sample.

We investigated differences in pneumococcal gene expression by examining RNA extracted directly from nasopharyngeal swabs and lung aspirates from the same 5 patients. After evaluation of RNA concentration and quality, samples containing serotype 3 were excluded from analysis due to DNA contamination, and samples containing serotype 14 were excluded due to insufficient RNA yield from the lung aspirate. Differences in expression of 9 genes in the lung aspirate compared with the nasopharynx are shown in Figure 2. Virulence genes *ply* (pneumolysin), *nanA* (neuraminidase A), and the putative virulence regulator *yeeN* were consistently and strongly upregulated (fold change >20) in the lung compared with the nasopharynx. In contrast, *psaB* (pneumococcal surface antigen B), *endA* (endonuclease), and the quorum sensing gene *luxS* were upregulated to a lesser extent, or varied by strain. Genes encoding pyruvate oxidase and enolase (s*pxB*, and *eno*, respectively) were downregulated in the lung, most prominently in serotype 1. Expression of *lytA* (autolysin) was downregulated in 12F but upregulated in serotypes 1 and 32A.

## DISCUSSION

We investigated the microbiological features of pneumococcal pneumonia using a unique set of clinical specimens obtained as part of pneumonia surveillance in The Gambia. This is a high pneumococcal carriage setting, reflected in the high colonization rates observed in both pneumonia patients and community controls. In children under 5 years of age, pneumococcal densities in the nasopharynx were over 1 log higher in pneumonia patients compared with controls. These results are consistent with data from Vietnam and the multicenter Pneumonia Etiology Research for Child Health (PERCH) study [2, 3]. Using whole genome sequencing, we demonstrated that pneumococci present in the lung of pneumonia patients originated from the nasopharynx. Although our study numbers were small, a previous study in The Gambia found the same serotype in the nasopharynx and lung for 23 of 27 (85%) pneumonia cases [1]. A multicenter study of children <5 years hospitalized for pneumonia reported that for 34 cases with pneumococci detected in blood, 27 (79%) had the same serotype detected in the nasopharynx [10]. These findings, taken together, provide further evidence that pneumococcal pneumonia originates from bacteria present in the upper respiratory tract, and they suggest that sampling the nasopharynx may provide some insight into pneumonia etiology.

A major finding from our study is that few DNA mutations were acquired by the pneumococcus during disease, whereas prominent differences in pneumococcal gene expression were observed between the lung and the nasopharynx. Although it is possible that mutations, for example in the *psaB* promoter region, may have impacted virulence, our results suggest that transcriptional differences rather than genomic changes underpin pneumococcal pathogenesis within the host. The *ply* gene, which encodes the pore-forming toxin pneumolysin, was strongly upregulated in the lung for all 3 isolates examined. Expression of *endA*, which encodes an endonuclease that degrades neutrophil extracellular traps (NETs), was upregulated in the lung, whereas *eno*, which encodes an enolase that can induce NET formulation, was downregulated [11, 12]. These results suggest that avoidance of NETs may facilitate pneumococcal survival in the lung. *SpxB* has been linked to multiple pneumococcal activities, including virulence, colonization, capsule synthesis, and transmission. Downregulation of s*pxB* in the lung may relate to one of these functions or its metabolic role in oxygen sensing and carbon source utilization [13]. It is interesting to note that the serotype 1 isolate displayed the most prominent changes in gene expression in the lung. This isolate belongs to sequence type 3081, a clone that is the leading cause of invasive pneumococcal disease in The Gambia and is highly virulent in animal models [14].

Viral testing was not conducted as part of this study. Not all lung aspirates underwent *lytA* qPCR, and serotyping was not conducted on the 5 culture-negative, *lytA*-positive lung aspirates. One key limitation is that gene expression studies were restricted to 3 paired specimens. We did not evaluate the expression of capsule, an important virulence factor. Certain capsular serotypes have been linked to pneumonia: serotypes 1, 5, 22F, 7F, and 14 were more likely to be found in the nasopharynx of children with pneumonia than in healthy controls in a study conducted before PCV use [15]. A similar study conducted in a post-PCV population would provide useful data for consideration of serotype coverage of next-generation PCVs. Although our sample size was small, data suggested a differing serotype distribution between pneumonia patients and community carriers, with serotypes 1 and 5 identified solely in patients.

## CONCLUSIONS

There is a paucity of data on pneumococcal gene expression from clinical samples, with 1 study to date examining pneumococcal RNA in the nasopharynx of healthy carriers [8]. Due to technical challenges, only 3 paired samples from our study were suitable for analysis of pneumococcal gene expression. Nevertheless, this study represents a proof-of-concept for conducting pneumococcal gene expression analysis directly from patient clinical specimens, identifying considerable changes in expression that occur within the lung. Although we hypothesize that the lung environment triggered these changes, other factors may have facilitated pneumococcal spread to the lung, and evaluating additional isolates is warranted. More important, specimens were stored in an RNA-stabilizing reagent promptly after collection. Future studies may expand upon the current findings by examining other sample types, such as sputum or pleural fluid, or applying transcriptomic approaches such as RNA sequencing (RNA-seq) to examine global changes in pneumococcal gene expression. In an experimental model, dual RNA-seq has been used to identify transcriptional changes in both pneumococci and the host associated with pneumococcal invasion into the pleural space [6]. The identification of molecular signatures associated with pneumococcal pneumonia could inform development of novel diagnostics and protein-based vaccines. We envision that future studies building upon this approach will extend our understanding of the pathogenesis of pneumococcal pneumonia.

## Supplementary Data

Supplementary materials are available at *The Journal of Infectious Diseases* online. Consisting of data provided by the authors to benefit the reader, the posted materials are not copyedited and are the sole responsibility of the authors, so questions or comments should be addressed to the corresponding author.

jiaa186_suppl_Supplementary_table_1

jiaa186_suppl_Supplementary_table_2

jiaa186_suppl_Supplementary_table_3

jiaa186_suppl_Supplementary_table_4

jiaa186_suppl_Supplementary_Material

## Notes

***Disclaimer*.** Funders had no role in study design, analysis, and interpretation of data, or manuscript preparation.

***Financial support*.** Pneumonia surveillance in The Gambia was funded by Gavi, the Vaccine Alliance, the Bill & Melinda Gates Foundation (OPP1020327), and the UK Medical Research Council. This study was funded by the Murdoch Children’s Research Institute, which was supported by the Victorian Government’s Operational Infrastructure Support Program. C. S. was supported by an Australian National Health Medical Research Council Career Development Fellowship (1087957) and a Veski Inspiring Women Fellowship. Y. H. was supported by a student research award from the Australian Society for Microbiology Victorian Branch.

***Potential conflicts of interest*.** C. S., E. M. D., and E. K. M. are investigators on an unrelated study funded by Pfizer. J. H. is an investigator on studies undertaken on behalf of St George’s, University of London or BUGS Bioscience that are sponsored or funded by vaccine manufacturers, including Pfizer, GlaxoSmithKline and Sanofi Pasteur. All authors have submitted the ICMJE Form for Disclosure of Potential Conflicts of Interest. Conflicts that the editors consider relevant to the content of the manuscript have been disclosed.

Presented in part: 2019 Australian Society for Microbiology Conference, June 30–July 3, 2019, Adelaide, Australia.
