# Supplementary material for: Insights Into Pneumococcal Pneumonia Using Lung Aspirates and Nasopharyngeal Swabs Collected From Pneumonia Patients in The Gambia
Source: J Infect Dis. 2020 Apr 22;225(8):1447–51. doi: 10.1093/infdis/jiaa186 (PMC9016440; doi:10.1093/infdis/jiaa186)
Supplement: jiaa186_suppl_Supplementary_table_1 [file jiaa186_suppl_supplementary_table_1.docx]

**Supplementary Table 1.** Primers used to evaluate pneumococcal gene expression by reverse transcriptase quantitative PCR.

| **Gene** | **Gene product/function** | **Primer sequence 5’ to 3’** | **Reference** |
| --- | --- | --- | --- |
| *ply* | Pneumolysin | F: CTACCCGATGAGTTTGTTGTT  R: TCCAGGATAGAGGCGACT | 1 |
|  |  |  |  |
| *luxS* | Quorum sensing | F: CACATGATTATGTGGGGACG  R: GGCATCATCTGAAATCCCTTG | This study |
|  |  |  |  |
| *nanA* | Neuraminidase A | F: CGTGGTTTGACTGGAGATC  R: CCATTTTCACGTTTCGGTCC | This study |
|  |  |  |  |
| *spxB* | Pyruvate oxidase | F: ATTCGGCGGCTCAATCGGGG  R: CAGCACGGCAGGCTTCGTCA | 1 |
| *gyrA* | Gyrase A (reference gene) | F: ATGGTCTCAAAGCGCTGAAT  R: TGGCGATACGACTCATACCA | 2 |
|  |  |  |  |
| *yeeN* | Putative virulence regulator | F: AAGGCTATCGTTGCTCTCC  R: TCTTTTGGACGTCTTCGTCG | This study |
|  |  |  |  |
| *eno* | Enolase | F: GACGGTACTCCTAACAAAGGTAAA  R: ATAGCTGTAAAGTGGGATTTCAA | 3 |
|  |  |  |  |
| *psaB* | Pneumococcal surface antigen | F: AGGAATGCGTCTCGTTAGGA  R: TAGTCAGCTAGGCCGACGAT | 4 |
|  |  |  |  |
| *lytA* | Autolysin | F: AGTTTAAGCATGATATTGAGAAC  R: TTCGTTGAAATAGTACCACTTAT | 3 |
| *endA* | Endonuclease | F: AGTCTGGAGTGGAATGGCTC  R: TCTTGACCTGATGCCAACCT | This study |

**References**

1. Cope EK, Goldstein-Daruech N, Kofonow JM, et al. Regulation of virulence gene expression resulting from *Streptococcus pneumoniae* and nontypeable *Haemophilus influenzae* interactions in chronic disease. PLoS One **2011**;6(12):e28523.

2. Pettigrew MM, Marks LR, Kong Y, et al. Dynamic changes in the *Streptococcus pneumoniae* transcriptome during transition from biofilm formation to invasive disease upon influenza A virus infection. Infect Immun **2014**;82(11):4607-19.

3. Sakai F, Talekar SJ, Klugman KP, et al. Expression of *Streptococcus pneumoniae* virulence-related genes in the nasopharynx of healthy children. PLoS ONE **2013**;8(6):1-9.

4. Heath CJ, del Mar Cendra M, Watson A, et al. Co-transcriptomes of initial interactions in vitro between *Streptococcus pneumoniae* and human pleural mesothelial cells. PLoS One **2015**;10(11):e0142773.
