# Supplementary material for: Insights Into Pneumococcal Pneumonia Using Lung Aspirates and Nasopharyngeal Swabs Collected From Pneumonia Patients in The Gambia
Source: J Infect Dis. 2020 Apr 22;225(8):1447–51. doi: 10.1093/infdis/jiaa186 (PMC9016440; doi:10.1093/infdis/jiaa186)
Supplement: jiaa186_suppl_Supplementary_table_2 [file jiaa186_suppl_supplementary_table_2.docx]

**Supplementary Table 2.** Participant characteristics for hospitalised pneumonia patients and community controls under 5 years of age who had nasopharyngeal swabs collected as part of pneumonia surveillance in The Gambia.

| **Characteristic** | | **Pneumonia patients**  (n=20) | **Community controls**  (n=22) | **P value** |
| --- | --- | --- | --- | --- |
| Age in months, median (IQR) | | 20 (16, 44) | 11 (8, 26) | 0.092 |
| Sex, n (%) | | | | |
|  | male | 12 (60) | 10 (45) | 0.346 |
|  | female | 8 (40) | 12 (55) |  |
| Received ≥ 2 doses of PCV13 | | 16 (80) | 20 (91) | 0.313 |
