# Supplementary material for: Insights Into Pneumococcal Pneumonia Using Lung Aspirates and Nasopharyngeal Swabs Collected From Pneumonia Patients in The Gambia
Source: J Infect Dis. 2020 Apr 22;225(8):1447–51. doi: 10.1093/infdis/jiaa186 (PMC9016440; doi:10.1093/infdis/jiaa186)
Supplement: jiaa186_suppl_Supplementary_table_3 [file jiaa186_suppl_supplementary_table_3.docx]

**Supplementary Table 3.** Multilocus sequence type (MLST) of paired pneumococcal isolates identified in the lung and nasopharynx of pneumonia patients.

| **Isolate** | **Site of isolation** | **serotype** | **MLST** | **NCBI accession number** |
| --- | --- | --- | --- | --- |
| BS7073F | nasopharynx | 3 | 11454 | SAMN14379526 |
| BS28919I | lung | 3 | 11454 | SAMN14379527 |
| BS6361F | nasopharynx | 14 | 2447 | SAMN14379528 |
| BS27990I | lung | 14 | 2447 | SAMN14379529 |
| BS7435F | nasopharynx | 12F | 989 | SAMN14379530 |
| BS29409I | lung | 12F | 989 | SAMN14379531 |
| BS7473 | nasopharynx | 32A | 15088^a^ | SAMN14379532 |
| BS29465I | lung | 32A | 15088^a^ | SAMN14379533 |
| BS7607F | nasopharynx | 1 | 3081 | SAMN14379534 |
| BS29645I | lung | 1 | 3081 | SAMN14379535 |

^a^Novel sequence type identified in this study
