# Supplementary material for: Insights Into Pneumococcal Pneumonia Using Lung Aspirates and Nasopharyngeal Swabs Collected From Pneumonia Patients in The Gambia
Source: J Infect Dis. 2020 Apr 22;225(8):1447–51. doi: 10.1093/infdis/jiaa186 (PMC9016440; doi:10.1093/infdis/jiaa186)
Supplement: jiaa186_suppl_Supplementary_table_4 [file jiaa186_suppl_supplementary_table_4.docx]

**Supplementary Table 4.** Single nucleotide polymorphisms and insertion/deletions identified by sequence comparison between nasopharynx and lung isolated from the same patient.

| **Serotype** | **gene** | **Gene product** | **Nucleotide change** | **Type of mutation** |
| --- | --- | --- | --- | --- |
| 3 | *rpoC* | RNA polymerase beta’ subunit | Single nucleotide substitution (202 C → T) | Missense mutation (R → C) |
| 1 | *glnA* | Glutamine  synthetase | Single nucleotide insertion (469 C) | Frame shift |
| 32A | *psaB* (promoter region) | Manganese transporter | Single nucleotide substitution (-18 C → A) | Nucleotide substitution in promoter |
