# Supplementary material for: Insights Into Pneumococcal Pneumonia Using Lung Aspirates and Nasopharyngeal Swabs Collected From Pneumonia Patients in The Gambia
Source: J Infect Dis. 2020 Apr 22;225(8):1447–51. doi: 10.1093/infdis/jiaa186 (PMC9016440; doi:10.1093/infdis/jiaa186)
Supplement: jiaa186_suppl_Supplementary_Material [file jiaa186_suppl_supplementary_material.docx]

**Detailed Methods**

**Study site and specimen collection**

This study was conducted in the Basse region of The Gambia. PCV7 was introduced nationally in 2009 and was replaced by PCV13 in 2011. Patients with suspected pneumonia had clinical assessment, blood culture, and chest x-ray performed. Lung aspiration was performed in patients with a dense, large, peripheral consolidation visible on chest x-ray, without any contraindications. Lung aspiration was performed in patients with a dense, large, peripheral consolidation radiologically without evidence of pneumatocoeles, the consolidation was accessible by transthoracic puncture and not adjacent to the heart, were clinically stable, had not recently had measles, and there was no skin infection at the puncture site. Lung aspiration is established as a safe practice in The Gambia, with an excellent safety record and high sensitivity as a diagnostic tool.[1] Percutaneous transthoracic lung aspiration was performed in a sterile manner with a 21 gauge needle attached to a 5 ml syringe containing 1 ml of sterile saline. The needle was inserted and withdrawn, maintaining maximal suction as the needle was being withdrawn over 2 seconds. The saline was flushed with the aspirated material into a sterile universal container. The specimen was transported immediately to the Medical Research Council laboratory at the Basse Field Station for examination by culture. A 100 µl aliquot was added to 200 µl RNAprotect Bacteria Reagent (Qiagen, Germany). Volumes are approximate as there was some variation across specimens. This aliquot and any remaining lung aspirate sample was then stored at -70˚C.

Nasopharyngeal swabs were collected from pneumonia patients and community controls according to World Health Organization recommendations using calcium alginate swabs placed in 1 ml skim milk-tryptone-glucose-glycerol media (STGG).[2] Community controls were identified in randomly selected households in age strata 2-23 months, 2-4 years, 5-14 years, and greater than or equal to 15 years. Nasopharyngeal swabs were transported to the Medical Research Council Field Station laboratory within 1 hour. A specimen for RNA work was prepared by adding 200 µl aliquot to 400 µl RNAprotect Bacteria Reagent (Qiagen, Germany) prior to storage along with the remaining STGG sample.

**Culture and *lytA* qPCR of lung aspirates**

Culture and bacterial identification were conducted as previously described.[3,4] In brief, promptly upon receipt to the Basse Field Station laboratory, lung aspirates were inoculated onto a 5% sheep blood agar, MacConkey agar, and Vitox (Oxoid) supplemented chocolate agar plates. Bacterial isolates were identified by standard systematic bacteriological methods, with pneumococci identified by colony morphology, optochin susceptibility, bile solubility, and reaction with polyvalent antisera (Statens Serum Institut, Copenhagen, Denmark).[3]

For detection of pneumococci by *lytA* real-time quantitative PCR (qPCR), lung aspirates were shipped on dry ice to the Medical Research Council microbiology laboratory in Fajara. DNA was extracted from 200 µl (or all remaining volume if less than 200 µl remained) using the QIAamp DNA Mini Kit from Qiagen, according to the manufacturer’s instructions. *LytA* qPCR was conducted as previously described[5], using a *lytA* plasmid standard curve. qPCR was conducted in 25 µl reactions containing 2.5 µl of template DNA on a CFX96 real time PCR instrument.

The laboratory methods described below were conducted at the Murdoch Children’s Research Institute in Australia following sample shipment on dry ice.

***lytA* qPCR and molecular serotyping of nasopharyngeal swabs**

DNA was extracted from 50 µl of STGG sample. After thawing, samples were pelleted by centrifugation for 10 min at 6,000 x g and incubated for 30 min at 37°C in an enzymatic solution containing 0.16 mg/ml lysostaphin and 3.1 mg/ml lysozyme, followed by a 2 min incubation at room temperature with 2.5 mg/ml RNase A (Qiagen). Extraction was completed using a MagNA Pure LC Machine (Roche) using the DNA Isolation Kit III (Bacteria, Fungi) (Roche).

Pneumococci were detected and quantified by *lytA* qPCR, using a standard curve of genomic DNA prepared from a reference strain of *S. pneumoniae*.[5] qPCR was conducted in 25 µl reactions containing 5 µl of template DNA on a Stratagene Mx3005 (Agilent Technologies) machine using Brilliant III Ultra, Fast qPCR Master Mix (Agilent Technologies). A cycle threshold (Ct) value < 35 was considered positive, and samples with Ct values from 35 - 40 were considered equivocal and later confirmed by culture. Pneumococcal density data were reported in genome equivalents/ml (GE/ml).

Samples were cultured on horse blood agar plates containing 5 µg/ml of gentamicin (Oxoid) prior to molecular serotyping by microarray. For samples with α-haemolytic growth, a representative colony was subcultured, tested for optochin sensitivity, and stored in STGG medium. The remainder of growth on the plate was harvested and DNA was extracted with the QIAcube HT instrument (Qiagen) and QIAamp 96 DNA QIAcube HT Kit (Qiagen), using a lysis buffer (20mM Tris/HCl, 2 mM EDTA, 1% v/v Triton, 20 mg/ml lysozyme) and RNase A treatment.[6] Molecular serotyping by microarray was performed using the Senti-SPv1.5 microarray (BUGS Bioscience) as previously described.[7]

**Whole genome sequencing and SNP validation**

Pneumococcal isolates from the lung and nasopharynx were serotyped by latex agglutination with confirmation by Quellung reaction when needed.[8,9] Pneumococcal DNA was extracted from isolates as previously described.^5^ DNA was sequenced in 2 x 300 bp paired end reads on the MiSeq platform. Using the Geneious 11.1.2 software package, sequence reads were trimmed with BBDuk and de novo assembled using SPAdes.[10] Single nucleotide polymorphisms (SNPs) and insertions/deletions were identified by mapping the reads of lung aspirate isolates to the genome assembly of their paired nasopharyngeal isolate using Geneious. Identified sequence variations were confirmed by Sanger sequencing following PCR amplification using KAPA HiFi HotStart ReadyMix PCR Kit (Promega, USA) and the following primers: *rpoC* (F 5'-GGGCTCATATCAAGGGTCAA-3', R 5'-TGGGAATGTGCTTGTGGTAA-3'), *psaB* (F 5'-TGCCAACGTTTCTTTGTAGG-3', R 5'-TCCATGATAAAACGCATAATTCC-3'), and *glnA* (F 5'-CTTCAACCTTGGTCCAGAGC-3', R 5'-CACAAGCACGGAGAACTTCA-3'). Following gel purification using the Wizard® SV Gel and PCR Clean-Up System (Promega), products were sequenced by the Australian Genome Research Facility using capillary separation on an AB 3730xl DNA analyser with BigDye™ Terminator v3.1 labelling.

**RNA extraction**

RNA was extracted from clinical samples previously stored in RNAProtect Bacteria Reagent (Qiagen). Thawed samples were diluted 1:2 with PBS to facilitate pellet formation, and centrifuged at 15,000 x g for 10 min at 4°C. Pellets were resuspended in 200 μl lysis buffer containing Tris-EDTA (TE) buffer, 15 mg/ml lysozyme, 0.075 mg/ml mutanolysin (Sigma-Aldrich), 2 mg/mL Proteinase K (Qiagen), and 1 U/μl SUPERaseIn™ RNase Inhibitor (Ambion), and incubated at 37°C for 10 min. Cells were further lysed by 350 μl Buffer RLT (Qiagen) (with 1% (v/v) β-mercaptoethanol), and mixed with 250 μl of absolute ethanol. Samples were then transferred to a spin column and extraction performed using the using the RNeasy Mini Kit (Qiagen) according to manufacturer’s instructions. An on-column DNase I treatment was performed with a 15 min incubation in DNase solution containing 218 Kunitz units DNase I (Qiagen) in Buffer RDD (Qiagen). RNA was eluted in 50 μl nuclease-free water. RNA concentration and integrity were measured using a 2200 Tape Station (Agilent Technologies, USA) with High Sensitivity RNA ScreenTape and Reagents (Agilent Technologies). RNA was stored at -80°C until further use.

**Reverse transcription qPCR (RT-qPCR)**

RNA was reverse transcribed into cDNA using iScript™ cDNA Synthesis Kit (Bio-Rad, USA) in 50 µl reactions, according to manufacturer’s instructions. As RNA extracted from clinical specimens varied in concentration, the amount of RNA added in each cDNA reaction was adjusted according to availability. For lung aspirates, 2.5 or 5.0 ng of RNA was added; for nasopharyngeal swabs, either 2.5, 5.0, or 10.0 ng were added. Reactions were conducted using T100 Thermal Cycler (Bio-Rad) with an incubation at 25°C for 5 min, followed by 20 min at 46°C, and 1 min at 95°C. Synthesised cDNA was stored at 4°C until further use.

RT-qPCR was conducted using the GoTaq qPCR System (Promega) in accordance with the MIQE guidelines.[11] Each 25 μL RT-qPCR reaction contained approximately 2 ng cDNA template, GoTaq qPCR Master Mix, 0.03 μM each of forward and reverse primers (Sigma-Aldrich); Supplementary Table 1. Reactions were run in duplicate wells using the Mx3005P qPCR instrument under the following cycling conditions: one cycle at 95°C for 2 min; 40 cycles at 95°C for 15 sec and 60°C for 1 min; and a dissociation curve analysis at the end. Relative fold change in gene expression was calculated using the 2^-ΔΔCt^ method, with expression levels of each gene normalised to *gyrA*.[12] No-reverse transcriptase control assays to detect DNA contamination were performed by conducting cDNA synthesis reactions without RT enzyme, followed by RT-qPCR targeting *ply* and *gyrA*. Samples containing serotype 3 were excluded from further analysis as DNA was detected.

**Acknowledgements**

We thank study participants and staff of the MRC Basse Field Station for supporting this study. We thank the MRC laboratory in Fajara for conducting qPCR on lung aspirates, and Elina Garcia Rodriguez and the MCRI pneumococcal microbiology laboratory for nasopharyngeal swab examination. We acknowledge Rieza Aprianto, Jan-Willem Veening, Christopher McDevitt, and Jason Rosch for advice on RNA extraction protocols.

**References**

1. Ideh RC, Howie SRC, Ebruke B, et al. Transthoracic lung aspiration for the aetiological diagnosis of pneumonia: 25 years of experience from The Gambia. Int J Tuberc Lung Dis **2011**;15(6):729-35. doi: 10.5588/ijtld.10.0468

2. Satzke C, Turner P, Virolainen-Julkunen A, et al. Standard method for detecting upper respiratory carriage of *Streptococcus pneumoniae*: updated recommendations from the World Health Organization Pneumococcal Carriage Working Group. Vaccine 2013;32(1):165-79.

3. Mackenzie GA, Hill PC, Jeffries DJ, et al. Effect of the introduction of pneumococcal conjugate vaccination on invasive pneumococcal disease in The Gambia: a population-based surveillance study. Lancet Infect Dis **2016**;16(6):703-11.

4. Adegbola RA, Falade AG, Sam BE, et al. The etiology of pneumonia in malnourished and well-nourished Gambian children. Pediatr Infect Dis J **1994**;13(11):975-82.

5. Carvalho Mda G, Tondella ML, McCaustland K, et al. Evaluation and improvement of real-time PCR assays targeting *lytA*, *ply*, and *psaA* genes for detection of pneumococcal DNA. J Clin Microbiol **2007**;45(8):2460-6. doi: 10.1128/JCM.02498-06

6. Manna S, Dunne EM, Ortika BD, et al. Discovery of a *Streptococcus pneumoniae* serotype 33F capsular polysaccharide locus that lacks wcjE and contains a wcyO pseudogene. Plos One **2018**;13(11):e0206622. doi: 10.1371/journal.pone.0206622

7. Satzke C, Dunne EM, Porter BD, et al. The PneuCarriage Project: a multi-centre comparative study to identify the best serotyping methods for examining pneumococcal carriage in vaccine evaluation studies. PLoS Med **2015**;12(11):e1001903

8. Porter BD, Ortika BD, Satzke C. Capsular serotyping of *Streptococcus pneumoniae* by latex agglutination. J Vis Exp **2014**(91):51747.

9. Habib M, Porter BD, Satzke C. Capsular serotyping of *Streptococcus pneumoniae* using the Quellung reaction. J Vis Exp **2014**(84):e51208.

10. Kearse M, Moir R, Wilson A, et al. Geneious Basic: An integrated and extendable desktop software platform for the organization and analysis of sequence data. Bioinformatics **2012**;28(12):1647-49.

11. Bustin SA, Benes V, Garson JA, et al. The MIQE guidelines: minimum information for publication of quantitative real-time PCR experiments. Clin Chem **2009**;55(4):611-22.

12. Livak KJ, Schmittgen TD. Analysis of relative gene expression data using real-time quantitative PCR and the 2(-Delta Delta C(T)) Method. Methods **2001**;25(4):402-8.
